# The effects of public and private health care expenditure on health status in sub-Saharan Africa: new evidence from panel data analysis

**DOI:** 10.1186/2191-1991-2-22

**Published:** 2012-12-11

**Authors:** Jacob Novignon, Solomon A Olakojo, Justice Nonvignon

**Affiliations:** 1 Department of Economics, University of Ibadan, Ibadan, Nigeria; 2 Department of Health Policy, Planning & Management, School of Public Health, University of Ghana, Legon, Ghana

**Keywords:** Health care expenditure, Health status, Fixed effects, SSA

## Abstract

**Background:**

Health care expenditure has been low over the years in developing regions of the world. A majority of countries in these regions, especially sub-Saharan Africa (SSA), rely on donor grants and loans to finance health care. Such expenditures are not only unsustainable but also inadequate considering the enormous health care burden in the region. The objectives of this study are to determine the effect of health care expenditure on population health status and to examine the effect by public and private expenditure sources.

**Methods:**

The study used panel data from 1995 to 2010 covering 44 countries in SSA. Fixed and random effects panel data regression models were fitted to determine the effects of health care expenditure on health outcomes.

**Results:**

The results show that health care expenditure significantly influences health status through improving life expectancy at birth, reducing death and infant mortality rates. Both public and private health care spending showed strong positive association with health status even though public health care spending had relatively higher impact.

**Conclusion:**

The findings imply that health care expenditure remains a crucial component of health status improvement in sub-Saharan African countries. Increasing health care expenditure will be a significant step in achieving the Millennium Development Goals. Further, policy makers need to establish effective public-private partnership in allocating health care expenditures.

## Background

Improvement in human capital has been identified as a critical catalyst to economic growth and development in the macroeconomic literature [1]. Specifically, the neoclassical endogenous growth model posits that, growth in human capital (knowledge) impacts positively on output per worker in the long run [1]. Similarly, Grossman’s human capital model suggests that quality health significantly influences human capital development through the additional working time and utility derived from good health [2]. Good health does not only improve individuals’ consumption and production in the short run, but also improves returns from investments in productive activities in the long run [3]. Novignon et al. [4] provided evidence to show that poor health status has significant negative influence on both current and future welfare of households.

Adequate and efficient health related spending is widely considered as inevitable in the improvement of health status [5]. At the macro level, investment in health workforce and infrastructure is expected to improve health conditions and hence human capital of the population. However, in sub-Saharan Africa (SSA) and other developing regions where resources are relatively scarce, health expenditure has received less attention in government budgets [6].

For instance, Poullier et al. [7] estimated that the share of income that countries spend on health is greater for high income countries, with health spending as a share of gross domestic product (GDP) ranging from about 1.5% to 13%. While the highest shares are found in Europe and the Americas, Africa and some Asian regions contribute the lowest. Relative to their contribution of 19% to the world’s population, Poullier et al. [7] found that OECD countries alone contribute about 85% of the world’s total health spending. However, Africa’s 10% contribution to the world’s population relates to 3% of the world’s health spending.

While the concept of health spending may vary from one country to the other, Poullier et al. [8] and Poullier et al. [7] provide a classification of total health spending. Total health expenditure is considered as a summation of both public and private spending on all health related goods and services. The public outlays of expenditure are usually financed through social security contributions, various forms of taxation to various branches of government and from external sources, including grants and loans. On the other hand, the private outlays encompass private insurance premiums and prepaid schemes, mandated enterprise health expenditure, expenditure on health through non-profit health services and direct payments or out of pocket (OOP) expenditure on health goods, which includes co-payments as well as direct payments by uninsured individuals.

In SSA, public health expenditure is largely financed by resources from grants and loans [6], which may be due to the poor tax systems and social security structures. This explains the high levels of private health expenditure in these regions including increasing catastrophic (OOP) health expenditure in the face of high level of income poverty [9]. This may also explain the poor health infrastructure and workforce across the region as limited resources are assigned to the provision and maintenance of health related infrastructure [6]. Further, public health insurance systems are largely underdeveloped across the majority of countries in the region with most health care systems based on ability-to-pay [10].

The relationship between health care expenditure and health status has received some attention in developing regions. At the country level, Akinkugbe and Mohanoe [11] performed time series analysis using the error correction model (ECM) and found that in addition to public health care expenditure, the availability of physicians, female literacy and child immunization significantly influenced health outcomes in Lesotho. At the regional level, Anyanwu and Erhijakpor [5] in a panel data analysis and using a fixed effect model found that total health expenditures are a significant contributor to health outcomes with a 10% increase in total health care expenditure per capita resulting in 21% and 22% decrease in under-five and infant mortality rates respectively. Akinkugbe and Afeikhena [12] also provided evidence that the effect of health care expenditure as a ratio of GDP on life expectancy, under-five mortality and infant mortality is positive and significant in SSA, Middle East and North Africa.

Other studies have found no evidence that health related spending has any effect on health outcomes [13]. Filmer and Pretchett [14] provided evidence to show that while health care spending impact on child mortality, it is not the dominant driver of this health outcome. Factors such as education, technological change, income and cultural differences have been identified by some researchers as major drivers of health outcomes rather than health care spending [14-19]. Burnside and Dollar [20] also show that there exists no significant relationship between health care expenditure and change in infant mortality in low-income countries.

The empirical evidence presented above suggests that the exact relationship between health care spending and health outcomes is not clear, especially at the macro level. While some studies have shown significant positive or negative impact of health care spending on health outcomes [5] others did not find any significant relationship between the two [20]. As noted by Wagstaff and Cleason [21], the extent to which public health care spending influences health outcomes depend on the effectiveness of policies and institutions.

The current study is motivated by the inconclusive debate on the relationship between health expenditure and health outcomes with particular attention on SSA and the fact that none of the above studies sought to analyse the differential effects of public and private health spending in SSA. The purpose of the study is, therefore, two-fold, first is to investigate the impact of total health care spending on various health outcomes after controlling for country-specific demographic structures and economic conditions. Secondly, a differential analysis of public and private health care spending is performed. The following null hypotheses were tested; (1) there is no significant relationship between health spending and health outcomes in SSA, (2) there is no significant difference in the effects of public and private health spending on health outcomes. The first hypothesis is in line with Akinkugbe and Afeikhena [12]. However, the second hypothesis has received little attention in SSA. A similar hypothesis was tested by Berger and Messer [22] across 20 OECD countries.

### Brief regional profiles

Table 1 presents the trend and value of health care expenditure across various regions in the world. Table 1 show that SSA spends the lowest on health care per capita (Table 1). Relative to the Middle East and North Africa with health care expenditure per capita of US$322 in 2010, SSA accounts for US$85 of health care expenditure per capita. This represents an increase from US$32 in 2000 but significantly falls short of the world’s average of US$949 in 2010 (Table 1). Health expenditure per capita is highest in the North American region with an increase from US$4,446 in 2000 to US$8,050 in 2010.

**Table 1:** Health care expenditure trend across regions in the world

**Regions**	**OOP HE (% of private HE)**		**HE per capita (current US$)**		**HE private (% of GDP)**		**HE public (% of GDP)**		**HE total (% of GDP)**	
	**2000**	**2010**	**2000**	**2010**	**2000**	**2010**	**2000**	**2010**	**2000**	**2010**
SSA	52	67	32	85	4	4	2	3	6	6
Middle East and North Africa	87	79	166	322	2	2	3	3	5	5
OECD	39	66	2284	4366	4	4*	6	7*	10	13
North America	26	32	4446	8050	7	8*	6	8*	13	17
East Asia & Pacific	88	73	249	500	2	2*	5	4*	7	7
World	44	70	486	949	4	4*	5	6*	9	10

* 2009 figures are reported in the absence of 2010 figures. **HE** is health expenditure.

Source: World development indicators.

OOP health expenditure as percentage of private health expenditure increased in SSA (from 52% in 2000 to 67% 2010), relative to the world average of 44% in 2000 to 70% in 2010. Some developed regions also recorded increases in OOP health spending over the period (Table 1). However, this becomes a source of worry in regions where poverty levels are very high with impoverished population. Increasing direct health care spending will only worsen population welfare and increase poverty.

Further, public health expenditure as percentage of GDP in 2010 was 3% for SSA relative to the average for the world (6%), North America (8%), East Asia and Pacific (4%). Similarly, total health expenditure as percentage of GDP was 6% for SSA, which suggests poor performance against the average for the world (10%), East Asia and Pacific (7%) and North America (17%). This suggests that health related expenditures still remain major concerns in developing regions like SSA (Table 1).

Again, SSA compares poorly with other regions of the world in terms of population health-related indicators (Table 2). Relative to the world’s average of 70 years, life expectancy at birth in SSA was 54 years in 2010, an increase from 50 years in 2000. In 2010, OECD countries had the highest life expectancy at birth (79 years) followed by North America (78 years), East Asia and Pacific (73 years) and Middle East and North Africa (72 years).

**Table 2:** Population health indicators across regions in the world

**Region**	**Life expectancy at birth**		**Improved sanitation**		**Improved water source**		**Infant mortality rate**		**HIV prevalence rate**	
	**2000**	**2010**	**2000**	**2010**	**2000**	**2010**	**2000**	**2010**	**2000**	**2010**
SSA	50	54	28	31	55	61	94	76	6	5*
Middle East and North Africa	70	72	82	89	88	89	37	26	0	0*
OECD	77	79	96	98	98	99	11	7	0	0*
North America	77	78	100	100	99	99	7	6	0	1*
East Asia & Pacific	71	73	53	69	82	91	29	19		0*
World	67	70	56	62	83	88	52	41	1	1*

* 2009 figures are reported in the absence of 2010 figures.

Source: World development indicators.

Sanitation facilities and water source was well improved in North America with about 100% of the population having access in 2010. On the other hand, access to improved sanitation facilities and water source was 31% and 61% respectively in SSA, relative to 62% and 88% respectively for the world average. Again, relative to other regions and the world average, SSA was the worst performer in terms of Human Immunodeficiency Virus (HIV) prevalence with about 5% of the population between 15 and 49 years being infected with the virus in 2010 (Table 2).

## Methods

### Data and variables

The study pooled cross-section and annual time series data from 1995 to 2010 for 44 countries in sub-Saharan Africa.^a^ The data used in the empirical analysis were sourced from the World Bank, World Development Indicators (WDI) [9].

The study uses life expectancy at birth, infant mortality rate and death rate as health status indicators. Life expectancy at birth was measured in years as average life expectancy of male and female population, infant mortality rate is measured per 1,000 live births and death rate measured as crude death rate per 1,000 people. Life expectancy has been criticised as a less accurate measure of population health relative to mortality rates due to measurement errors [23]. Healthy life expectancy at birth represents a broader measure of population health as mortality, morbidity, disability and other health indicators are considered in its measurement [6]. However, life expectancy at birth reflects how many years a person might be expected to live given prevailing mortality rates. Using the three indicators of health outcome will, therefore, allow for robustness in the analysis.

While total, public and private health care expenditures are measured as percentage of GDP, Income per head is measured as GDP per capita at constant 2000 US$. Higher health care expenditure is expected to relate to higher life expectancy at birth and lower infant mortality and death rates. Finally, different population age groups namely, age below 14 years, 15 to 64 years and above 65 years were measured as percentage of total population. These were included to control for the different country demographic structures. Relative to the younger population, the population age group above 65 years is expected to reduce health outcomes by increasing death rates [23]. To control for the varying levels of health status in SSA, HIV prevalence rate was included in the model. The variable was measured as the percentage of population living with HIV.

### The model

The study starts with a health status model specified in a panel form as follows:

(1) $$y_{\mathrm{it}}=X_{\mathrm{it}}\beta +\varepsilon_{t},t=1.....T$$

(2) $$\varepsilon_{t}=\mathrm{\mu W}+\upsilon$$

Where *y*_*it*_ is a vector of dependent variables in country *i* at time *t*, *X* is a vector of exogenous variables, including the constant, and *β* is a vector of coefficients. ε_t_ is a vector of random error terms. Following Baltagi et al. [24], equation two decomposes the error process into a summation of two components; time variant and remainder error process. The error term is spatially correlated with the spatial weights matrix, *W*, and has spatial autocorrelation parameter *μ*.

For the purposes of this study, the following model specifications were estimated;

(3) $$HS_{\mathrm{it}}=\alpha_{i}+\beta_{1}HE_{\mathrm{it}}+\beta_{2}Y_{\mathrm{it}}+\beta_{4}\mathrm{POPU}1_{\mathrm{it}}+\beta_{5}\mathrm{POPU}2_{\mathrm{it}}+\beta_{6}\mathrm{POPU}3_{\mathrm{it}}+\varepsilon_{\mathrm{it}}$$

Where *HS* represents three health outcomes (Life expectancy at birth, Infant mortality rate and Death rate) in country *i* in period *t*. HE is the total health expenditure as percentage of real national income. *Y* is per capita real income which acts as a control variable for the demand for health services and other economic factors. The variables *POPU1, 2 and 3* represents population age groups of below 14, 15–64 and above 65 years respectively expressed as a percentage of total population. *α*_*i*_ is time invariant and captures country-specific effect that was not included in the model. The error terms were assumed to be normally distributed. ε_it_ is the error term.

Total health care expenditure is further grouped into private and public health care expenditure. This was to allow for the analysis of the individual impact of each of these components. The model in equation three was therefore re-specified as follows;

(4) $$HS_{\mathrm{it}}=\alpha_{i}+\beta_{1}\mathrm{PuH}E_{\mathrm{it}}+\beta_{2}\mathrm{Pr}HE_{\mathrm{it}}+\beta_{3}Y_{\mathrm{it}}+\beta_{5}\mathrm{POPU}1_{\mathrm{it}}+\beta_{6}\mathrm{POPU}2_{\mathrm{it}}+\beta_{7}\mathrm{POPU}3_{\mathrm{it}}+\varepsilon_{\mathrm{it}}$$

Where *PuHE* and *PrHE* represent public and private health care expenditure, respectively.

Random effects model was estimated by Generalized Least Squares (GLS) while fixed effects model was estimated by pooled least squares ordinary least squares via GLS (with cross-section weights). The fixed effect model was estimated in two forms, as a model assuming a single overall constant term for the cross sections (common intercept specification) and as a model assuming separate constant terms for the cross sections (with overall constant term). The most appropriate result was reported following the Hausman specification test. The Hausman specification test was carried out to choose between random effects and fixed effects models. While the results favoured results from the fixed effects model, both the fixed and random effect models were reported for comparison purposes and to allow for robustness of results.

Baltagi et al. [24] argued that in spatial econometrics, where the error term is considered not serially correlated with the remainder error and there is no spatial serial dependence of the error terms, the random effect estimation is more appropriate. Cameron and Trivedi^b^[25] also observed that fixed effects may be used to control for endogeneity in panel data where endogeneity arises owing to a time-invariant omitted variable. The E-views statistical software package was used in the analysis.

## Results

### Descriptive statistics

Table 3 shows that average total health care expenditure as percentage of GDP in SSA was estimated to be approximately 10%. Average public and private health care expenditure were estimated to be about 3% and 6% of GDP, respectively. While life expectancy at birth had a mean of about 52 years, infant mortality per 1000 live births and crude death rates per 1000 people had estimated means of 84 and 14, respectively. Approximately 7% and 14% of the population had access to improved sanitation facilities and improved water source, respectively. On average, hospital bed per 1000 population was about 1. While the population between the ages 0 and 14 years was 43% on average, about 54% and 3% were within the age groups of 15–64 years and above 65 years, respectively (Table 3).

**Table 3:** Descriptive statistics

**Variable**	**Mean**	**Std dev**	**Min**	**Max**
Health care expenditure (total)	9.71	5.51	1	17
Health care expenditure (public)	3.32	1.81	1	9
Health care expenditure (private)	5.97	4	1	15
Infant mortality	84.23	27.77	13	162
Crude death rate	14.29	4.41	5	38
Life expectancy	52.06	7.09	27	74
GDP per capita	270.17	150.44	1	566
HIV prevalence	11.98	9.97	1	29
OOP health expenditure	29.79	25.33	1	88
Improved sanitation	7.08	14.65	1	63
Improved water source	13.86	25.25	1	93
Hospital beds	1.34	0.87	1	6
Age below 14	43.26	4.16	22	49
Age 15-64	53.58	3.6	48	71
Age above 65	3.16	0.82	2	7

### Health expenditure and life expectancy at birth

Results from the fixed and random effects models are reported in Tables 4, 5 and 6 for life expectancy at birth, death rate and infant mortality rate respectively. Table 4 shows that increase in total health expenditure (as% of GDP) was more likely to increase life expectancy at birth at 1% significance level. A 1% increase in total health expenditure leads to an improvement in life expectancy at birth by approximately 0.7 years in the fixed effects model and about 0.6 years in the random effects model (Table 4).

**Table 4:** Effects of health care expenditure on life expectancy at birth (years)

	**GLS-fixed effects model**		**GLS-random effects model**	
**Variables**	**(1)**	**(2)**	**(1)**	**(2)**
Constant	41.577 (1.351)	42.261 (1.364)	41.357 (1.347)	43.000 (1.390)
Real GDP Per Capita	0.002 (5.647)***	0.002 (4.516)***	0.002 (5.474)***	0.002 (4.548)***
Health Expenditure (Total)	0.697 (7.032)***		0.615 (6.486)***	
Health Expenditure (Public)		1.039 (5.401)***		0.983 ( 5.198)***
Health Expenditure (Private)		0.528 (4.148)***		0.443 (3.697)***
Population >14 yrs	0.041 (0.136)	0.024 (0.078)	0.051 (0.169)	0.024 (0.082)
Population 15-64 yrs	0.103 (0.325)	0.104 (0.325)	0.111 (0.351)	0.102 (0.319)
Population >65 yrs	−0.421 (−1.161)	−0.491 (−1.342)	−0.433 (−1.198)	−0.509 (−1.393)
HIV Prevalence rate	−0.204 (−3.763)***	−0.162 (−2.683)**	−0.175 (−3.517)***	−0.148 (−2.740)**
R-squared	0.735	0.734	0.138	0.139
Durbin-Watson	0.173	0.161	0.142	0.136
F-Stat.	21.705***	20.952***	11.180***	9.502***
Observations	424	120	424	421
Cross section included	43		43	43

**Note:** ***significant at 1%; **significant at 5%; *significant at 10%.

t-statistics are reported in parenthesis.

**(1)** is model with aggregate health care expenditure.

**(2)** is model with aggregate health expenditure decomposed into public and private.

**Table 5:** Effects of health care expenditure on death rate (per 1000 people)

	**GLS-fixed effects model**		**GLS-random effects model**	
**Variables**	**(1)**	**(2)**	**(1)**	**(2)**
Constant	17.587 (0.786)	17.645 (0.782)	17.778 (0.796)	17.070 (0.759)
Real GDP Per Capita	−0.001 (−4.947)***	−0.001 (−3.874)***	−0.001 (−4.629)***	−0.001 (−3.763)***
Health Expenditure (Total)	−0.567 (−7.865)***		−0.505 (−7.359)**	
Health Expenditure (Public)		−0.839 (−5.991)***		−0.797 (−5.812)***
Health Expenditure (Private)		−0.445 (−4.812)***		−0.381 (−4.416)***
Population <14 yrs	0.018 (0.080)	0.025 (0.115)	0.010 (0.045)	0.026 (0.115)
Population 15-64 yrs	−0.036 (−0.158)	−0.042 (−0.182)	−0.042 (−0.184)	−0.040 (−0.175)
Population >65 yrs	0.377 (1.429)	0.422 (1.586)	0.386 (1.467)	0.436 (1.642)
HIV Prevalence rate	0.125 (3.172)**	0.099 (2.244)**	0.10 (2.803)**	0.085 (2.182)**
R-squared	0.703	0.702	0.148	0.151
Durbin-Watson	0.211	0.204	0.176	0.174
F-Stat.	18.508***	17.904***	12.148***	10.53
Observations	424	421	424	421
Cross section included	43	43	43	43

**Note:** ***significant at 1%; **significant at 5%; *significant at 10%.

t-statistics are reported in parenthesis.

**(1)** is model with aggregate health care expenditure.

**(2)** is model with aggregate health expenditure decomposed into public and private.

**Table 6:** Effects of health care expenditure on infant mortality rate (per 1000 live births)

	**GLS-fixed effects model**		**GLS-random effects model**	
**Variables**	**(1)**	**(2)**	**(1)**	**(2)**
Constant	126.324 (1.003)	128.033 (1.007)	127.442 (1.013)	127.097 (1.001)
Real GDP Per Capita	−0.000 (−0.104)	0.001 (0.580)	−0.000 (−0.075)	0.001 (0.480)
Health Expenditure (Total)	−3.005 (−7.411)***		−2.834 (−7.237)***	
Health Expenditure (Public)		−4.204 (−5.328)***		−3.965 (−5.100)***
Health Expenditure (Private)		−2.478 (−4.748)***		−2.322 (−4.678)***
Population <14 yrs	−0.119 (−0.095)	−0.093 (−0.074)	−0.147 (−0.119)	−0.105 (−0.083)
Population 15-64 yrs	−0.480 (−0.370)	−0.527 (−0.403)	−0.509 (−0.392)	−0.535 (−0.409)
Population >65 yrs	2.579 (1.737)*	2.787 (1.857)*	2.600 (1.754)*	2.814 (1.878)*
HIV Prevalence rate	−0.330 (−1.482)*	−0.452 (−1.820)	−0.340 (−1.641)	−0.416 (−1.836)*
R-squared	0.762	0.762	0.155	0.142
Durbin-Watson	0.139	0.135	0.12	0.116
F-Stat.	25.049***	24.192***	12.751***	9.757***
Observations	424	421	424	421
Cross section included	43	43	43	43

**Note:** ***significant at 1%; **significant at 5%; *significant at 10%.

t-statistics are reported in parenthesis.

**(1)** is model with aggregate health care expenditure.

**(2)** is model with aggregate health expenditure decomposed into public and private.

Disintegrating the effect of total health expenditure shows that an increase in both public and private health care expenditure significantly (at 1% level) increased life expectancy at birth by about 1 and 0.5 years, respectively, in the fixed effects model and about 1 and 0.4 years, respectively, in the random effects model (Table 4).

### Health expenditure and death rate

Table 5 shows that an increase in total health expenditure reduces death rate (per 1000 people) by approximately 0.6 in the fixed effects model and 0.5 in the random effects model with a significance level of 1%. While public health care expenditure reduced death rate by about 0.8 in both fixed and random effects models, private health care expenditure reduced death rate by approximately 0.4 per 1000 people in the fixed and random effects models, respectively, at 1% significance level (Table 5).

### Health expenditure and infant mortality rate

Total health care expenditure was more likely to reduce infant mortality rate (per 1000 live births) with 1% level of significance (Table 6). A 1% increase in total health expenditure reduced infant mortality rate by approximately 3 infants per 1000 live births in both the fixed and random effects models, respectively. While public health care expenditure reduced infant mortality rate by approximately 4 infants in both models at 1% significance level, an increase in private health care expenditure by 1% reduced infant mortality rate by 2 infants per 1000 live births in both models at 1% significance level (Table 6).

## Discussion

The findings of the study suggest that increasing health care spending remains an important step in improving health outcomes in sub-Saharan Africa. The results show that total health care expenditure, whether public or private, significantly improves the life expectancy at birth in sub-Saharan African countries. Similarly, total health care expenditure, irrespective of the source, significantly reduces the number of deaths per 1000 people and infant mortality rate per 1000 live births.

The findings were expected as health expenditure, especially public, are used for providing and developing health facilities and improving health system operations. This conforms with findings of other studies that health expenditure is an important determinant of health outcomes both at the individual and national levels [22,26,27]. For instance, Or [28] found significant positive relationship between spending on health and health outcomes. On the contrary, some studies have found that the influence of health expenditure on health status is either small or statistically insignificant [13,14,29].

In SSA, where health infrastructure is largely underdeveloped, increasing health care expenditure will be a significant progress towards improving health outcomes and accelerating progress towards the health-related Millennium Development Goals (MDGs). It must be noted that while the findings of the current study provides evidence in support of increasing health care expenditure, this may only be a necessary but not sufficient condition as achieving progress in terms of population health may depend on the effective and efficient allocation of such resources. It is possible for population health to worsen even as health care expenditure increase in the face of misallocation and poor management.

The results suggest that even though both public and private health care expenditure had similar relationship with life expectancy, death and infant mortality rates, the relative impact of the two sources of health expenditure were different. Public health care expenditure was found to have higher impact on all the three measures of health outcomes relative to private health care expenditure. This was expected as a majority of health facilities in SSA are public owned and funded. In this regard, public health expenditure is more likely to impact on a greater proportion of the population than private health expenditure. However, this does not conform with the findings of Berger and Messer [22] that increases in publicly financed share of health expenditures are associated with increases in mortality rates. They noted that their result was robust to a number of specifications and samples and concluded that “as countries increase the level of their health expenditure, they may want to reduce the proportion of their expenditures that are publicly financed”.

On the contrary, the current study argues that the relationship is positive for SSA in the sense that the health sector in this region is faced with goods and services which are likely to be provided in inefficient quantities if left to the private sector. The participation of the public sector is therefore critical in ensuring that such goods and services are provided in equitable and efficient quantities so as to improve the health of the population. These goods and services include the provision of health care infrastructure, training of health personnel, immunization and other preventive health care measures. Further, the findings of Berger and Messer [22] may not hold in the case of SSA due to the high levels of poverty and impoverished standards of living in the region. In this regard, private health care spending (e.g. out of pocket) will only worsen the poverty situation.

The findings of the study also underscore the role of health care expenditure in achieving the health related MDGs. Thus, as shown by the magnitude of the coefficients, increase in health care expenditure will not only improve population health but also infant health which is an important component of the health related MDGs.

The study is limited in the sense that most of the health outcome variables for SSA did not have enough time series observation which would have improved a panel data study as this one. The proxies used in measuring health outcomes may not be exhaustive as such information as morbidity and disability are not captured. Including three different health outcome measures, however, allows for robustness of results. While these limitations may be the bases for future research, they do not invalidate the results of the current study.

## Conclusion

The study sought to determine the impact of health care expenditure on health status measured by life expectancy at birth, crude death rate and infant mortality rate in SSA. The results provided evidence that health care expenditure was associated with increase in life expectancy at birth and reduction in death and infant mortality rates. The results also showed that while both private and public sources of health care expenditure were significantly associated with improved health outcomes, public health care expenditure had relatively larger impact.

The findings imply that, health care expenditures are essential components in improving health status in SSA. There is need for governments in the region to increase amounts allocated to health care service delivery. In addition, establishing effective public-private partnerships in developing the health sector could go a long way to improve population health status.

## Endnotes

^a^The following countries were included in the study: Angola, Benin, Burkina Faso, Botswana, Burundi, Cameroon, Cape Verde, Central African Republic, Chad, Comoros, Congo Demographic Republic, Cote d’Ivoire, Equatorial Guinea, Eritrea, Ethiopia, Gabon, Ghana, Guinea, Guinea Bissau, Kenya, Lesotho, Liberia, Madagascar, Malawi, Mali, Mauritania, Mauritius, Mozambique, Namibia, Niger, Nigeria, Rwanda, South Africa, Senegal, Sierra Leone, Somalia, Sudan, Swaziland, Tanzania, The Gambia, Togo, Uganda, Zambia and Zimbabwe.

^b^Cameron and Trivedi (2005) Capter 23, Page 801

## Competing interests

The authors declare that they have no competing interests.

## Authors’ contributions

JN (1) and SAO conceived the study, undertook the analysis. JN(2) contributed to refining the conception. JN(1), JN(2) and SAO wrote and reviewed the manuscript. All authors read and approved the final manuscript.
